# Mortality from ischaemic heart disease by country, region, and age: Statistics from World Health Organisation and United Nations^☆^

**DOI:** 10.1016/j.ijcard.2012.10.046

**Published:** 2013-09-30

**Authors:** Judith A. Finegold, Perviz Asaria, Darrel P. Francis

**Affiliations:** a International Centre for Circulatory Health, National Heart and Lung Institute, London, UK; b Department of Epidemiology and Biostatistics, Imperial College School of Public Health, Imperial College London, UK

**Keywords:** Ischaemic heart disease, Coronary heart disease, Mortality, Trends

## Abstract

**Background:**

Ischaemic heart disease (IHD) is the leading cause of death worldwide. The World Health Organisation (WHO) collects mortality data coded using the International Statistical Classification of Diseases (ICD) code.

**Methods:**

We analysed IHD deaths world-wide between 1995 and 2009 and used the UN population database to calculate age-specific and directly and indirectly age-standardised IHD mortality rates by country and region.

**Results:**

IHD is the single largest cause of death worldwide, causing 7,249,000 deaths in 2008, 12.7% of total global mortality. There is more than 20-fold variation in IHD mortality rates between countries. Highest IHD mortality rates are in Eastern Europe and Central Asian countries; lowest rates in high income countries. For the working-age population, IHD mortality rates are markedly higher in low-and-middle income countries than in high income countries.

Over the last 25 years, age-standardised IHD mortality has fallen by more than half in high income countries, but the trend is flat or increasing in some low-and-middle income countries. Low-and-middle income countries now account for more than 80% of global IHD deaths.

**Conclusions:**

The global burden of IHD deaths has shifted to low-and-middle income countries as lifestyles approach those of high income countries. In high income countries, population ageing maintains IHD as the leading cause of death. Nevertheless, the progressive decline in age-standardised IHD mortality in high income countries shows that increasing IHD mortality is not inevitable. The 20-fold mortality difference between countries, and the temporal trends, may hold vital clues for handling IHD epidemic which is migratory, and still burgeoning.

## 1 Introduction

Ischaemic heart disease (IHD) is the leading cause of death worldwide [1–4], placing a major economic and resource burden on health and public health systems. High income countries have seen declines in mortality rates from IHD [5–10], but elsewhere the picture is less favourable, with continued high IHD mortality [11]. Reliable information describing time-trends in IHD mortality is essential to understand and monitor the disease [12]. In this article we provide an overview of the global epidemiology of IHD mortality using data submitted by individual member states to the World Health Organisation. The data cover the period 1995 to 2009, and are more complete for some countries than for others. We have analysed the data to allow identification of country-specific and broad regional trends.

**Fig. 1:**
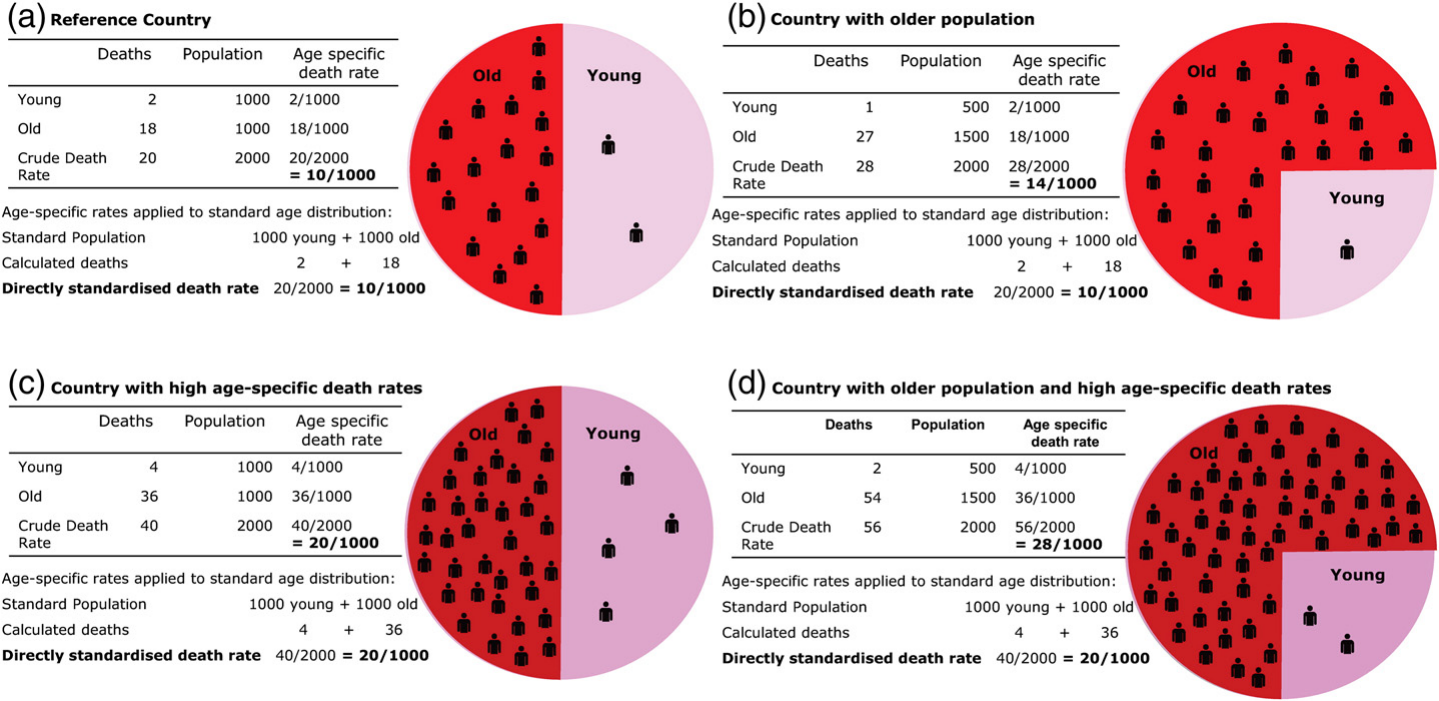
(a) A standard population of 2000 people, distributed equally amongst "young" (under 60 years) and "old" (60 years and older) groups which have different death rates. This distribution of ages will be used as the "standard" in the other panels. (b) A country with the same age-specific risks, but whose population is older. Crude death rate is higher because a greater proportion of people are in the high-risk age group. However, age-standardisation prevents the ageing artefact by reconstituting a population of the "standard" age distribution, to obtain the same standardised death rate as (a). (c) A country which, compared with (a), has double the death rate at each age group. Crude death rates, and age-standardised death rates are doubled (d) A country with double the age-specific mortality and an older population. Crude mortality is very much higher but age-standardised mortality, which reconstitutes a standard distribution of ages, is only twice that of panel (a).

**Fig. 2:**
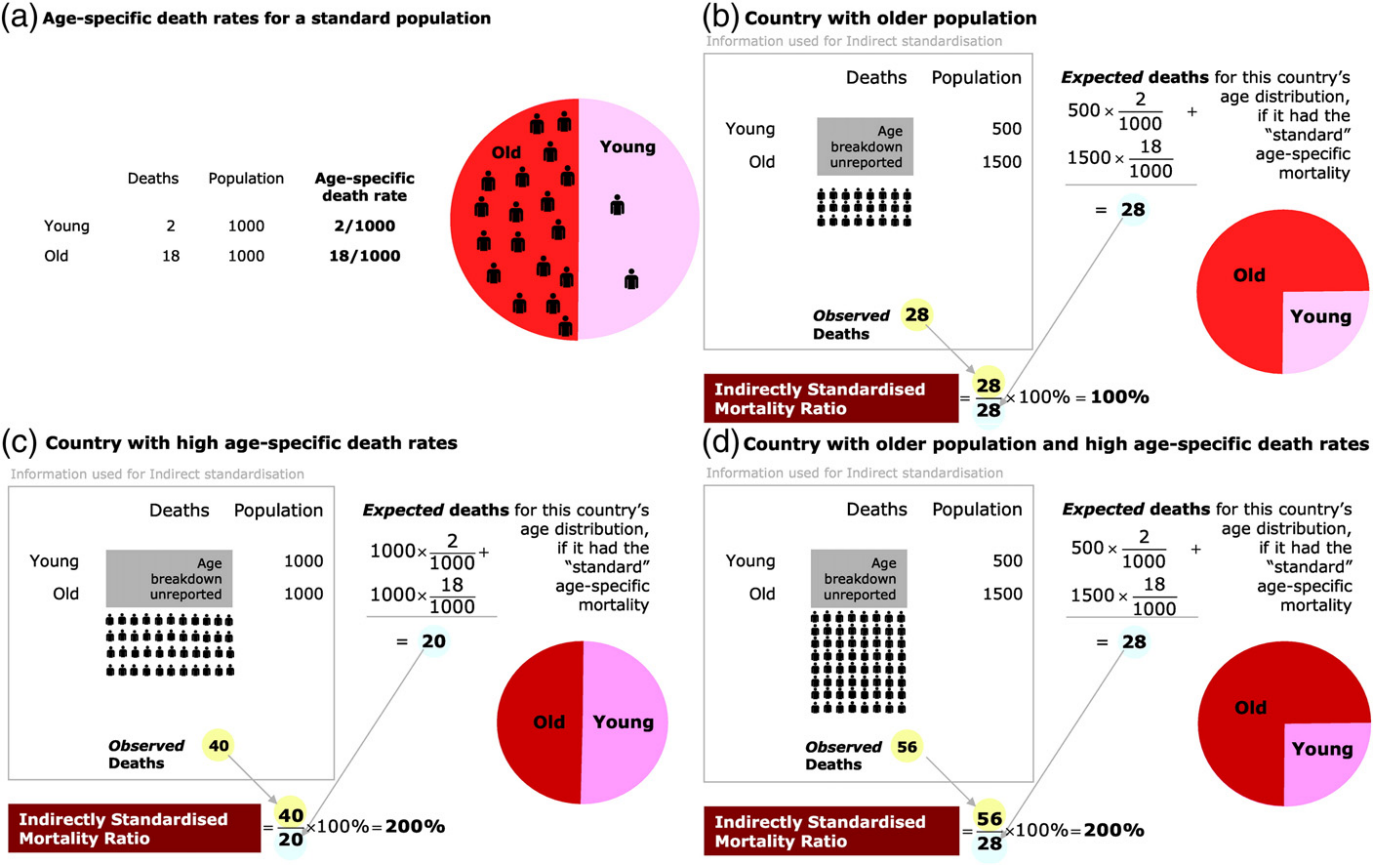
If a country reports age-specific population but only total IHD deaths (without an age breakdown), it is not possible to calculate directly standardised mortality rates. Instead, by making the assumption that the age relationship of mortality is a scaled-up or scaled-down version of that of a standard population, it is possible to calculate an indirectly standardised mortality ratio expressing the country's mortality relative to that of the standard population. Panels (b), (c) and (d) calculate the indirectly standardised mortality ratio for the same country data as the corresponding panels in Figure 1, but with the age breakdown of deaths concealed.

We present absolute IHD burden, along with directly and indirectly standardised IHD mortality rates. Absolute burden reflects the total number of deaths a health system has to deal with, which will tend to be larger in more populous countries. Standardised rates are preferable for comparing countries because they remove the effects of population size and age structure. The most comprehensive standardisation is direct standardisation (Fig. 1), which requires age-specific data for both the number of deaths in each country and its population size. In many countries age-specific data on deaths from IHD are not available, but the UN does provide modelled estimates of age-specific population counts for all countries. Indirect standardisation (Fig. 2) divides the deaths observed in a country, by the deaths expected if that country had the same age-specific death rates as a population group chosen to be the standard for comparison. We present indirectly standardised ratios for countries where direct standardisation is not possible due to lack of age-specific death data.

## 2 Methods

### 2.1 Data sources

IHD mortality data (ICD9 codes 410–414 and ICD10 codes I20–25) between 1995 and 2009 were extracted from the online World Health Organisation (WHO) mortality database [13] and from WHO publications [14,15]. These data comprise all deaths registered by national civil registration systems which were submitted to WHO, with underlying cause of death coded by the relevant national authority using the International Statistical Classification of Diseases and Related Health Problems (ICD) 9th or 10th revision [16]. In some countries data were only available for parts of this time period: in these cases the available years are shown. Population data were from UN population estimates also available online [17]. For comparisons by region and country income group, we have used 2001 data from the WHO, which are the most complete.

### 2.2 Statistics

#### 2.2.1 Age-specific and age-standardised mortality rates

Age specific mortality rates for each five year age group are presented. In addition we present directly standardised IHD mortality rates (standardised to WHO world standard population) [18] to allow comparison of mortality rates between countries. We also present indirectly standardised ratios for countries where direct standardisation is not possible, by calculating deaths expected if a country had the same age-specific death rate as the standard population (defined here as the average age-specific mortality rates in 2001 of the UK, USA, Canada, France and Germany).

The authors of this manuscript have certified that they comply with the principles of ethical publishing in the International Journal of Cardiology.

These authors take responsibility for all aspects of the reliability and freedom from bias of the data presented and their discussed interpretation.

## 3 Results

### 3.1 Burden of IHD worldwide in 2008

In 2008 there were 7,249,000 deaths from IHD, accounting for 12.7% of all global deaths. India and China together had over 2 million deaths or over 30% of the world's total IHD burden (Table 1). Large numbers of deaths were also seen in the Russian Federation (659,000) and in USA (445,800) reflecting their large population sizes.

Worldwide in 2001 [15], IHD was the leading cause of death in both low-and-middle income countries (11.8% of all deaths) and high income countries (17.3% of all deaths). At that time IHD was the leading cause of death in all but two world regions: Sub-Saharan Africa and East Asia and the Pacific. IHD was the leading cause of death in Europe and Central Asia (29.7% of total deaths), Middle East and North Africa (16.9% of total deaths), South Asia (13.6% of total deaths) and Latin America (10.9% of total deaths). In contrast, in Sub-Saharan Africa IHD was the eighth cause of death after HIV/AIDS, malaria, lower respiratory tract infections, diarrhoeal disease, perinatal conditions, measles, cerebrovascular disease, accounting for only 3.2% total deaths. In East Asia and the Pacific, IHD was the third leading cause of death accounting for 8.8% of total deaths.

### 3.2 Impact of age on IHD mortality

**Fig. 3:**
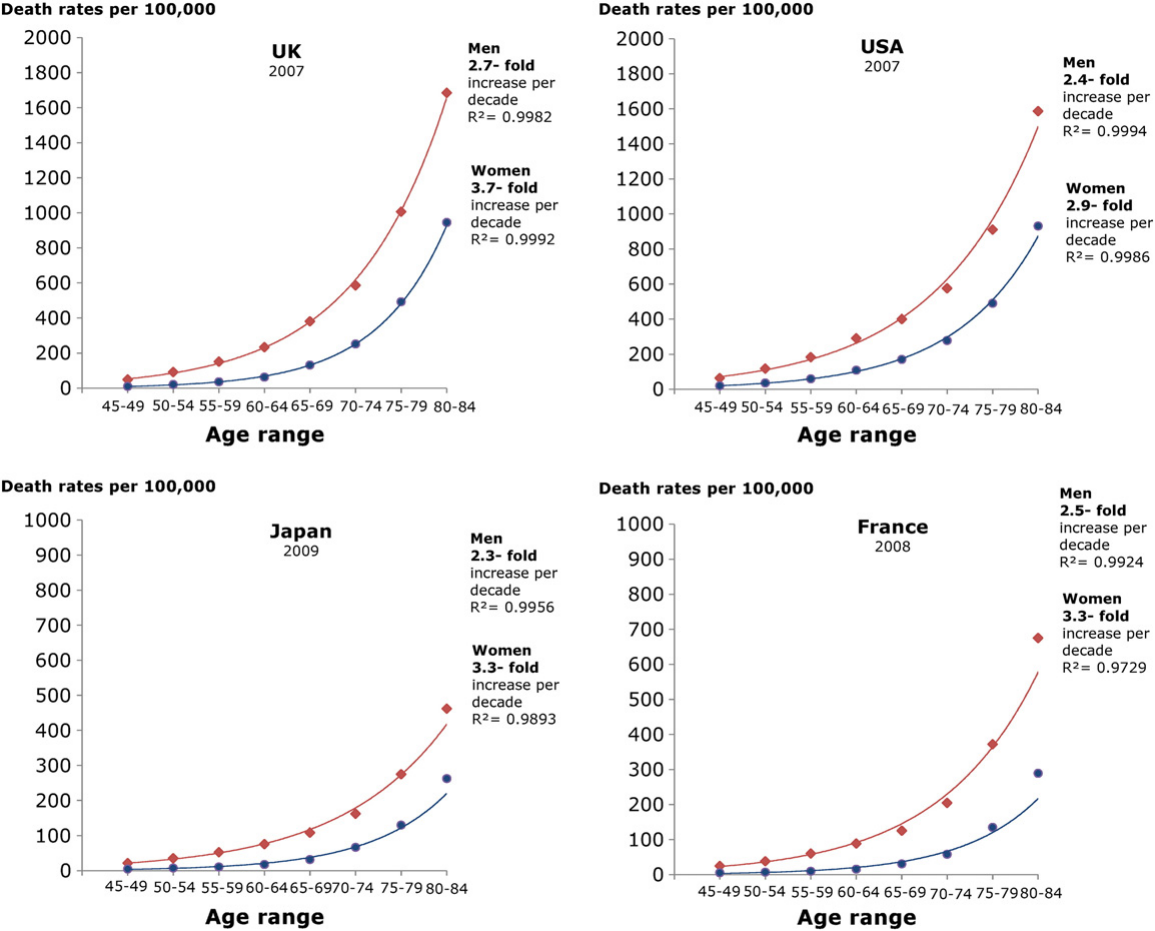
Change in mortality with age in UK, USA, Japan and France.

**Fig. 4:**
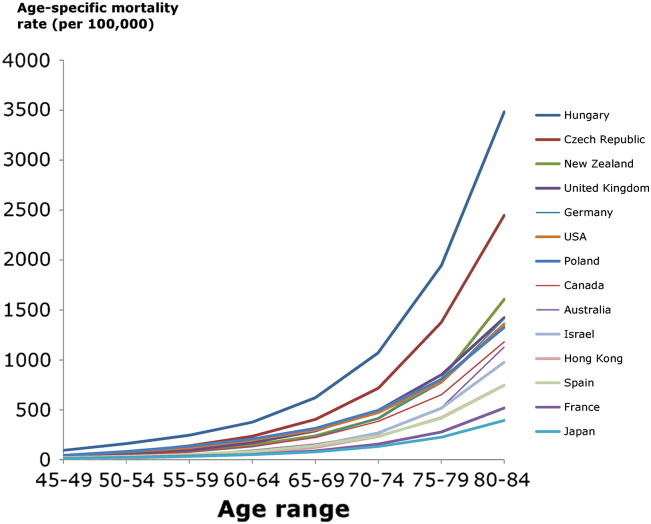
IHD mortality by population age group for selected countries. Age-specific mortality rate for selected countries. The most recent year of available data between 2005 and 2009 is displayed.

Fig. 3 shows the progressive increase in IHD mortality with age in 4 selected countries: UK, USA, Japan and France. In the years illustrated, women have a lower IHD mortality rate than men. There is a progressive increase in IHD mortality with age, which fits an exponential trend. In these countries, chosen for illustration because they have complete data, there is a 2.3 to 2.7-fold increase in IHD mortality for every decade of life for men and a 2.9 to 3.7-fold increase for women. Fig. 4 illustrates that the exponential rise in mortality with age is present in all countries regardless of starting mortality level or country income level.

### 3.3 Age specific mortality rates from IHD in 2001

Age specific mortality rates for IHD were higher in low-and-middle income countries than in high income countries (Tables 2a and 2b). For older ages highest rates were seen in Europe and Central Asia, South Asia and the Middle East and North Africa. For example, for men in the age range 80 years and over the death rates per 100,000 population were 8598 in Europe and Central Asia, 3758 in Middle East and North Africa and 3644 in South Asia. Meanwhile in high income countries the rate was 2253 per 100,000 population.

The difference in mortality between regions was even more marked for premature IHD mortality in the working-age population (defined as those aged < 60 years). In men this was again highest in Europe and Central Asia and then Middle East and North Africa and South Asia. For example, in men in the age range 45–59 years the death rate in Europe and Central Asia was 517 per 100,000 population, in Middle East and North Africa 304 and in South Asia 302. By contrast in high income countries, the IHD death rate in the same age group was 91 per 100,000 population, less than one-fifth of the mortality seen in low-and-middle income Europe and Central Asia.

### 3.4 Time trends in age-standardised IHD mortality rates

Time series of directly-standardised IHD mortality rates between 2000 and 2009 (standardised to the WHO world standard population) are shown for selected countries with available data in Table 3.

**Fig. 5:**
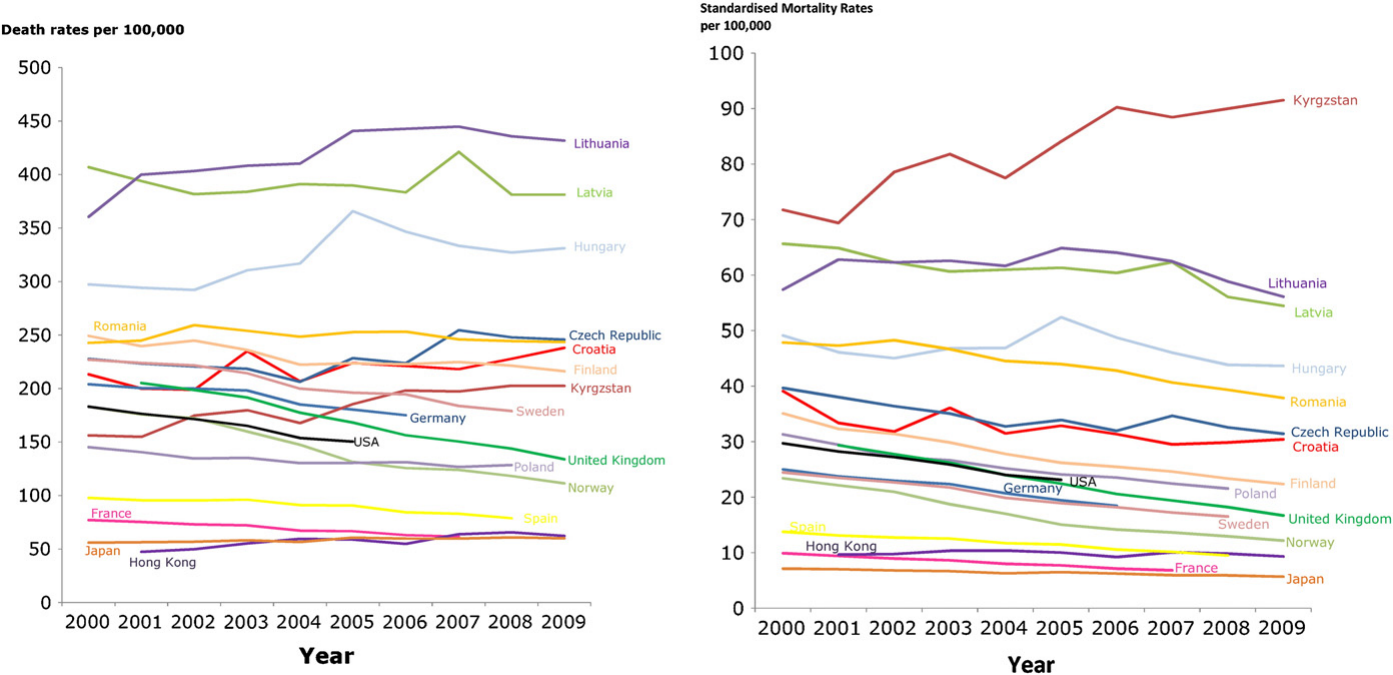
(a). Changes in crude annual mortality rates from ischaemic heart disease for selected Countries between 2000 and 2009. (b). Changes in directly standardised annual mortality rates from ischaemic heart disease for selected Countries between 2000 and 2009.

Age-standardisation is essential when comparing countries, as shown in Fig. 5a and b which contrast the crude and age-standardised mortality trends amongst the same set of countries. A decrease in age-standardised mortality rates is seen in most countries in Western Europe, but countries in Eastern Europe have a flat pattern (Croatia, Serbia, Slovakia, Hungary and Czech Republic) and an increasing trend can be seen in some Central Asian countries (e.g. Kyrgyzstan).

In the majority of countries for which age-specific data are available, there has been a steady decline in IHD death rates between 2000 and 2009. These data tend to be for high income countries. Reporting of time trends in IHD mortality is limited for most low-and-middle income countries.

### 3.5 Extended analysis across 177 countries: standardised mortality ratios

In order to compare a more comprehensive set of low, middle and high income countries that do not have complete age-specific IHD death data, we use indirectly standardised IHD mortality ratios presented in Table 4. Countries are ranked by their 2008 IHD mortality ratio. The spectrum is wide, with some countries such as Turkmenistan, Afghanistan and Ukraine having a mortality ratio that is approximately 20-fold that of other countries such as Japan.

## 4 Discussion

IHD has been, and continues to be, the single largest cause of death in the world. This is because the majority of the world's population lives in low-and-middle income countries, where IHD mortality rates are often flat or increasing, and total populations are growing.

Overall, age-standardised mortality has fallen significantly in many high income countries since the early 1980s [19]. However, the age effect on IHD mortality is so strong that high income countries, which have older and ageing populations, have a total mortality burden which remains high and is falling only slowly over time.

The larger populations and higher age-specific death rates for IHD in low-and-middle income countries mean that they already account for the majority of global IHD deaths and will bear the brunt of the IHD epidemic in the years to come. Moreover the combined effect of population growth and ageing is so strong that despite all current efforts, total numbers of IHD deaths worldwide are increasing.

Of the many factors that contribute to the favourable trend in IHD mortality in high income countries, three may be particularly important [20]. Firstly, policy changes may favour risk factor modification, such as decreased exposure to tobacco smoke [21] and improvement in primary prevention strategies [22,23] (e.g. hypertension control [24–26]). Second, rapid response times and improved treatments (such as thrombolysis and primary angioplasty) for acute IHD events may lead to reduced IHD case-fatality [27]. Third, secondary prevention, which may further reduce mortality, is making more headway in implementation in wealthier countries [28].

The contribution of these factors to the decreasing mortality rates from IHD is complex. The WHO MONICA (MONitoring trends and determinants In CArdiovascular disease) study [29,30] reported that between the mid-1980s and 1990s, on average two thirds of the decline in mortality from IHD could be attributed to a decline in coronary event rates and one third to decreasing case-fatality. A more recent study by Smolina et al. [31] during the 2000s in England reported just over half of the decline in IHD mortality could be attributed to a decline in event rates and just less than half to improved survival at thirty days.

The increasing mortality in some countries in Eastern Europe is likely to reflect a combination of continued high exposure to cardiovascular risk factors (including tobacco smoke [32]) and inadequate prevention strategies e.g. poor control of hypertension [33,34]. In addition, evidence suggests a positive association between excess alcohol consumption in Eastern European countries and increased mortality from cardiovascular disease [35]. Unfortunately, data are sparse for many areas of the world e.g. Latin America, Africa that are likely to be incurring continued unfavourable trends in IHD mortality due to rapid urbanisation and the shifting focus of tobacco companies and processed food and drinks manufacturers to low-and-middle income regions [36]. Rapid urbanisation has been positively associated with risk factors related to IHD [37] e.g. smoking, high BMI, poor blood pressure control and lower physical activity. Without accurate baseline mortality data it will be difficult to target prevention strategies for the future.

## 5 Limitations

A major limitation to our analysis of world-wide IHD trends is the paucity of data from certain geographical areas such as Latin America and Africa. The majority of data currently reported to the WHO is from high income countries with poor representation from low-and-middle income countries.

Despite the existence of the WHO ICD coding system which tries to standardise cause of death coding, there may still be differences in reporting patterns between countries, for example in the handling of deaths where multiple causes may have contributed, or where background medical information is scarce. In addition the validity of the ICD code assigned may vary from place to place. This may lead to either over or under-reporting of death due to ischaemic heart disease. In addition, the uncertainty around cause of death coding may be exaggerated in low-and-middle income countries, as some large countries such as India and China have not implemented fully comprehensive death registration systems but rely on sample surveillance for vital statistics [38,39].

Although low-and-middle income countries will struggle more than high income countries to find resources to target IHD prevention, it is in these countries that the impact could be greatest as they have the highest age-specific death rates in younger people. IHD contributes substantially to premature mortality in these populations, and reducing IHD mortality will increase the lifespan of working-age populations, and may therefore have an important effect on economic growth [40]. Not all preventative strategies will be prohibitively expensive. For example generic aspirin and statins are available cheaply, and government initiatives to reduce smoking need not be financially demanding [41]. While we acknowledge that many low-and-middle income countries may have competing healthcare priorities, this paper highlights the burden of IHD, and the critical need to target preventative and treatment interventions for IHD in these areas. It is particularly important that we do not hastily assume intervention in these countries would be futile, since they now bear the majority of the global burden of IHD deaths [42,43]. The impact of prevention and treatment strategies has been projected for non-communicable diseases in low-and-middle income countries and can potentially reduce age standardised death rates by 2% per year [44].

## 6 Conclusion

This paper illustrates mortality trends from IHD between 1995 and 2009 derived from WHO international data. IHD mortality rises sharply with age and IHD thus remains the leading cause of death even in high income countries where age-standardised death rates have fallen by as much as 50% in the last 25 years, as their populations age. In addition, premature mortality (at age < 60 years) from IHD is far from trivial, and age-specific death rates in the working age population are much higher in low-and-middle income countries than high income countries. This, combined with the large number of persons at risk in this age group in low-and-middle income countries, makes IHD a particular challenge for these countries.

Rising trends in IHD mortality are not inevitable as the declining age-standardised mortality rates in many high income countries demonstrate. Reliable information is the first step to successful initiatives to tackle this potentially tractable but currently uncontrolled epidemic. The opportunities are great because — unlike many other causes of death — the susceptibility of IHD to prevention and treatment is exquisite.

## Figures and Tables

**Table 1:** Global burden of IHD deaths (thousands).

High income countries			East Asia and Pacific			Europe and Central Asia			Latin American and the Caribbean			Middle East and North Africa			South Asia			Sub-Saharan Africa		
Rank	Country	Number	Rank	Country	Number	Rank	Country	Number	Rank	Country	Number	Rank	Country	Number	Rank	Country	Number	Rank	Country	Number
1	USA	445.8	1	China	1040.6	1	Russian Federation	659.5	1	Brazil	133.9	1	Iran	88.0	1	India	1248.0	1	Nigeria	71.6
2	Germany	155.8	2	Indonesia	242.8	2	Ukraine	338.1	2	Mexico	77.0	2	Egypt	78.8	2	Pakistan	195.5	2	Ethiopia	43.5
3	Japan	105.5	3	Myanmar	58.2	3	Turkey	81.2	3	Argentina	36.4	3	Morocco	33.7	3	Bangladesh	163.4	3	Sudan	39.2
4	United Kingdom	92.3	4	Philippines	57.8	4	Romania	56.7	4	Colombia	27.7	4	Iraq	26.7	4	Afghanistan	30.9	4	Dem Republic Congo	27.0
5	Italy	87.8	5	Thailand	53.3	5	Belarus	56.1	5	Venezuela	21.2	5	Yemen	17.7	5	Nepal	21.1	5	South Africa	20.5
6	Poland	79.0	6	Malaysia	22.7	6	Uzbekistan	51.7	6	Cuba	18.2	6	Algeria	14.7	6	Sri Lanka	15.9	6	Tanzania	19.1
7	Spain	43.5	7	Cambodia	9.1	7	Kazakhstan	48.3	7	Chile	9.8	7	Syrian Arab Republic	14.3	7	Bhutan	0.9	7	Côte d'lvoire	14.9
8	France	42.6	8	Lao People's Republic	5.7	8	Bulgaria	23.1	8	Dominican Republic	9.7	8	Tunisia	10.1	8	Maldives	0.1	8	Uganda	13.5
9	Canada	42.0	9	Papua New Guinea	4.5	9	Georgia	20.0	9	Peru	9.2	9	Libyan Arab Jamahiriya	6.7				9	Kenya	13.5
10	Hungary	33.6	10	Mongolia	1.1	10	Serbia	17.5	10	Honduras	6.2	10	Lebanon	5.9				10	Ghana	13.1
11	Czech Republic	28.5	11	Fiji	0.8	11	Azerbaijan	16.3	11	El Salvador	4.9	11	Jordan	4.7				11	Mozambique	12.9
12	Australia	24.9	12	Timor-Leste	0.5	12	Republic of Moldova	16.2	12	Ecuador	4.4	12	Djibouti	0.8				12	Cameroon	11.3
13	Saudi Arabia	20.9	13	Solomon Islands	0.2	13	Lithuania	14.9	13	Guatemala	4.4							13	Malawi	9.4
14	Republic of Korea	20.5	14	Samoa	0.1	14	Kyrgyzstan	12.9	14	Uruguay	4.0							14	Angola	7.6
15	Slovakia	18.4	15	Vanuatu	0.1	15	Turkmenistan	11.7	15	Paraguay	3.1							15	Madagascar	7.4
16	Sweden	17.0	16	Tonga	0.1	16	Armenia	10.5	16	Jamaica	3.1							16	Somalia	7.1
17	Austria	14.6				17	Latvia	9.9	17	Nicaragua	3.1							17	Zambia	6.9
18	Belgium	13.9				18	Tajikistan	6.7	18	Haiti	2.9							18	Guinea	5.7
19	Greece	13.1				19	Bosnia and Herzegovina	5.5	19	Costa Rica	2.8							19	Chad	5.3
20	Netherlands	12.7				20	Albania	5.4	20	Panama	1.7							20	Zimbabwe	5.2
21	Croatia	12.3				21	Montenegro	0.6	21	Guyana	0.9							21	Burkina Faso	5.1
22	Finland	11.4							22	Suriname	0.4							22	Benin	4.1
23	Portugal	10.4							23	St Vincent and Grenades	0.1							23	Mali	4.0
24	Switzerland	9.8							24	Belize	0.1							24	Niger	3.7
25	Denmark	6.7							25	Saint Lucia	0.1							25	Burundi	3.6
26	Norway	6.0							26	Grenada	0.1							26	Senegal	3.5
27	New Zealand	5.7																27	Rwanda	3.4
28	Ireland	5.3																28	Togo	2.9
29	Estonia	4.9																29	Republic	2.8
30	Singapore	4.7																30	Congo	2.1
31	Israel	4.7																31	Sierra Leone	1.8
32	Slovenia	2.4																32	Liberia	1.6
33	Oman	2.2																33	Eritrea	1.5
34	Trinidad and Tobago	1.7																34	Mauritius	1.4
35	Kuwait	1.2																35	Lesotho	1.4
36	United Arab Emirates	1.2																36	Namibia	1.3
37	Cyprus	1.0																37	Mauritania	1.2
38	Malta	0.7																38	Guinea-Bissau	1.0
39	Luxemberg	0.5																39	Botswana	0.8
40	Iceland	0.4																40	Gabon	0.8
41	Equatorial Guinea	0.4																41	Swaziland	0.7
42	Bahrain	0.2																42	Gambia	0.7
43	Qatar	0.2																43	Comoros	0.3
44	Barbados	0.2																44	Cape Verde	0.2
45	Bahamas	0.1																45	Principe	0.1
46	Brunei Darussalam	0.1																		

**Table 2a:** Age specific death rates from IHD in 2001 (Male).

Regions	Age specific death rates from IHD (per 100,000 population)							
	0–4	5–14	15–29	30–44	45–59	60–69	70–79	80 +
Low income countries								
East Asia and Pacific	1	1	3	15	79	304	779	1606
Europe and Central Asia	0	0	6	89	517	1591	3571	8598
Latin America and Caribbean	0	0	3	17	126	414	939	1956
Middle East and North Africa	0	0	5	45	304	956	2156	3758
South Asia	2	2	5	35	302	1005	2207	3644
Sub-Saharan Africa	0	0	1	14	139	526	1345	2291
High income countries								
High income countries	0	0	1	13	91	298	805	2253

**Table 2b:** Age specific death rates from IHD in 2001 (Female).

	Age Specific Death Rates from IHD (per 100,000 population)							
Regions	0–4	5–14	15–29	30–44	45–59	60–69	70–79	80 +
Low income countries								
East Asia and Pacific	0	0	3	8	47	227	647	1776
Europe and Central Asia	0	0	2	16	132	666	2261	7911
Latin America and Caribbean	0	0	1	7	55	223	567	1758
Middle East and North Africa	0	0	2	16	137	587	1565	3618
South Asia	2	1	9	25	163	790	1945	3217
Sub-Saharan Africa	0	0	1	6	86	410	1041	2212
High income countries								
High income countries	0	0	0	3	23	107	401	1789

**Table 3:** Age-standardised IHD mortality for selected countries between 2000 and 2009. Data directly standardised to WHO world standard population.

	Standardised mortality rates from ischaemic heart disease (per 100,000 population)									
	2000	2001	2002	2003	2004	2005	2006	2007	2008	2009
Australia	21.7	20.4	18.9	17.7	16.6		13.5			
Austria			23.7	21.8	19.9	19.4	18.8	18.5	17.1	17.3
Bulgaria						34.3	31.8	29.1	27.0	
Canada	22.9	21.3	20.0	19.5	18.1					
Croatia	39.1	33.4	31.8	36.1	31.5	32.8	31.3	29.5	29.8	30.4
Cyprus					16.7	17.6	15.9	18.5	15.7	
Czech Republic	39.7	38.0	36.4	35.1	32.7	33.9	31.9	34.7	32.5	31.4
Denmark	21.8	21.7	18.8	17.5	16.2	14.7	13.5			
Egypt					14.9	17.3	22.6	23.4	20.1	
Estonia	67.8	66.3	63.1	59.9	55.1	50.4	48.8	46.1	42.0	
Finland	35.1	32.3	31.4	29.8	27.8	26.2	25.5	24.6	23.3	22.4
France	9.9	9.4	8.9	8.6	8.0	7.7	7.1	6.8		
Germany	25.0	23.7	22.9	22.3	20.7	19.4	18.4			
Hong Kong		9.6	9.7	10.3	10.4	10.0	9.2	10.1	9.8	9.3
Hungary	49.1	46.1	45.0	46.8	46.9	52.4	48.7	46.0	43.8	43.6
Iceland	23.1	21.2	22.3	19.3	21.6	17.5	16.2	16.8	16.4	
Israel	18.8	16.8	15.3	15.0	12.9	12.4	12.2	12.5		
Japan	7.1	7.0	6.8	6.7	6.3	6.5	6.2	6.0	5.9	5.7
Kuwait	26.4	27.9	27.0					25.3	25.2	30.2
Kyrgyzstan	71.8	69.4	78.6	81.8	77.5	84.1	90.2	88.4	90.0	91.5
Latvia	65.6	64.9	62.3	60.7	61.0	61.3	60.4	62.3	56.1	54.5
Lithuania	57.4	62.8	62.3	62.6	61.7	64.9	64.0	62.5	58.9	56.1
Luxembourg	18.3	16.4	15.9	18.7	16.1	13.3	15.4	13.1	12.1	
Malta	35.4	33.9	30.8	30.9	26.5	30.2	27.9	24.0	24.7	
Netherlands	18.9	17.2	16.2	15.4	13.5	12.6	11.3	10.3	9.6	8.9
New Zealand	27.5	27.7	26.1	24.2	23.8	20.8	20.1	19.1		
Norway	23.4	22.1	20.9	18.7	17.0	15.0	14.1	13.6	12.9	12.2
Poland	31.3	29.4	27.2	26.6	25.2	24.1	23.5	22.4	21.5	
Romania	47.8	47.3	48.3	46.6	44.5	44.0	42.8	40.6	39.3	37.9
Serbia	28.7	28.0	28.7	28.7	28.1	31.4	30.4	27.9	27.3	26.5
Slovakia	59.0	53.9	52.4	53.2	52.5	50.8				
Slovenia	20.8	20.0	17.6	18.3	16.5	16.0	14.0	13.9	14.4	13.3
Spain	13.7	13.1	12.7	12.5	11.7	11.5	10.6	10.1	9.5	
Sweden	24.5	23.5	22.7	21.7	19.9	18.9	18.2	17.2	16.5	
United Kingdom		29.3	27.7	26.2	24.0	22.4	20.6	19.4	18.2	16.7
USA	29.7	28.2	27.2	25.9	24.0	23.1				

**Table 4:**
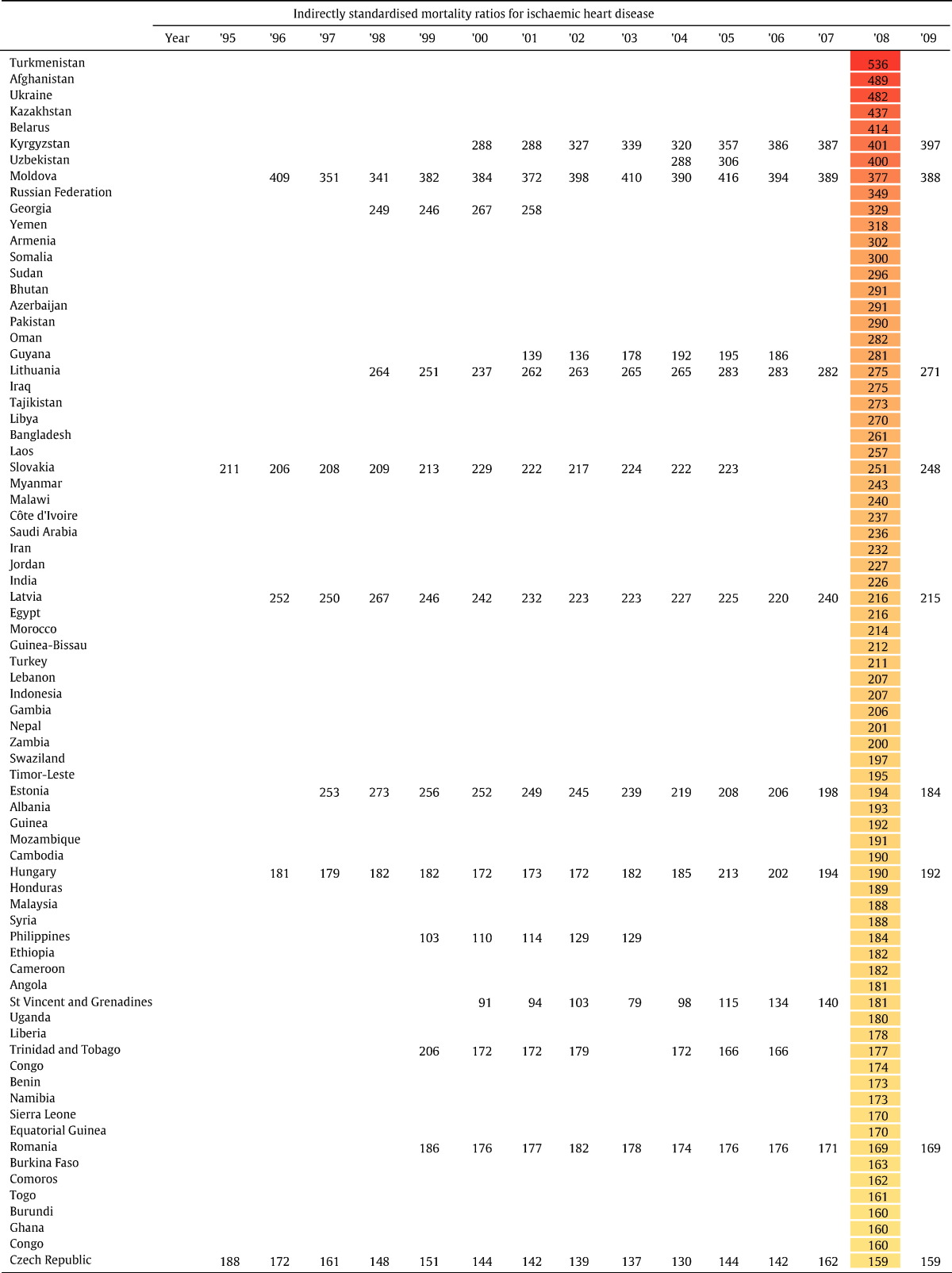

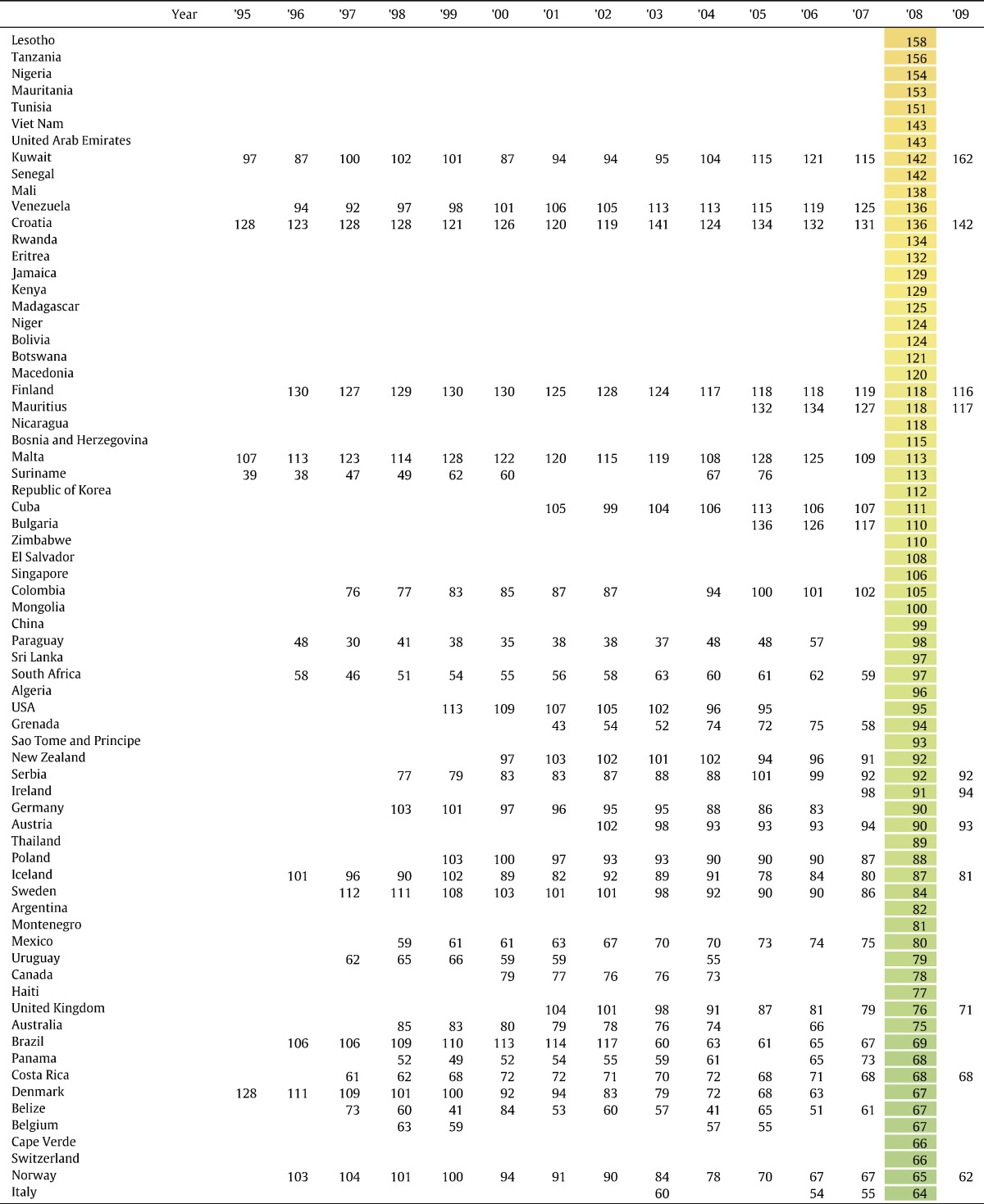

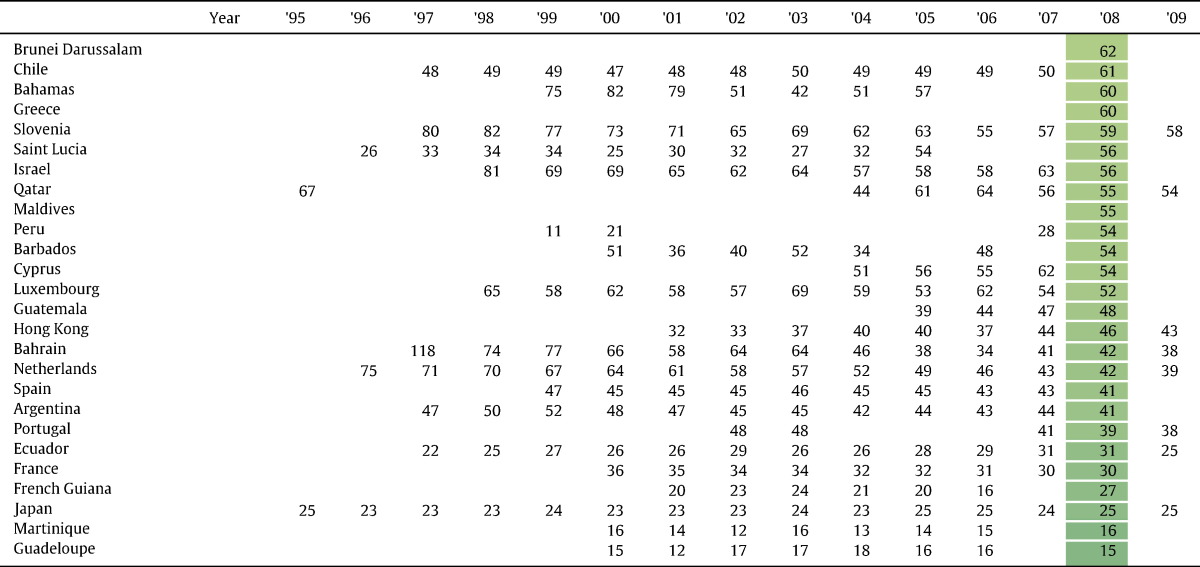
Indirectly standardised mortality ratios (SMR)^a^ for IHD for all WHO member states ranked by country SMR in 2008.

a Standard population is defined as the average age-specific mortality rates of the UK, USA, Canada, France and Germany in 2001.
